# Evaluation of Lysis Methods for the Extraction of Bacterial DNA for Analysis of the Vaginal Microbiota

**DOI:** 10.1371/journal.pone.0163148

**Published:** 2016-09-19

**Authors:** Christina Gill, Janneke H. H. M. van de Wijgert, Frances Blow, Alistair C. Darby

**Affiliations:** 1 Institute of Infection & Global Health, University of Liverpool, 8 West Derby Street, Liverpool, Merseyside, L69 7BE, United Kingdom; 2 Institute of Integrative Biology and the Centre for Genomic Research, University of Liverpool, Biosciences Building, Crown Street, Liverpool, Merseyside, L69 7ZB, United Kingdom; Argonne National Laboratory, UNITED STATES

## Abstract

**Background:**

Recent studies on the vaginal microbiota have employed molecular techniques such as 16S rRNA gene sequencing to describe the bacterial community as a whole. These techniques require the lysis of bacterial cells to release DNA before purification and PCR amplification of the 16S rRNA gene. Currently, methods for the lysis of bacterial cells are not standardised and there is potential for introducing bias into the results if some bacterial species are lysed less efficiently than others. This study aimed to compare the results of vaginal microbiota profiling using four different pretreatment methods for the lysis of bacterial samples (30 min of lysis with lysozyme, 16 hours of lysis with lysozyme, 60 min of lysis with a mixture of lysozyme, mutanolysin and lysostaphin and 30 min of lysis with lysozyme followed by bead beating) prior to chemical and enzyme-based DNA extraction with a commercial kit.

**Results:**

After extraction, DNA yield did not significantly differ between methods with the exception of lysis with lysozyme combined with bead beating which produced significantly lower yields when compared to lysis with the enzyme cocktail or 30 min lysis with lysozyme only. However, this did not result in a statistically significant difference in the observed alpha diversity of samples. The beta diversity (Bray-Curtis dissimilarity) between different lysis methods was statistically significantly different, but this difference was small compared to differences between samples, and did not affect the grouping of samples with similar vaginal bacterial community structure by hierarchical clustering.

**Conclusions:**

An understanding of how laboratory methods affect the results of microbiota studies is vital in order to accurately interpret the results and make valid comparisons between studies. Our results indicate that the choice of lysis method does not prevent the detection of effects relating to the type of vaginal bacterial community one of the main outcome measures of epidemiological studies. However, we recommend that the same method is used on all samples within a particular study.

## Introduction

The microbes that inhabit various niches of the human body have the potential to significantly affect the health of their host [1]. For instance, studies of the vaginal microbiome have shown that certain types of microbiota are associated with a reduced risk of acquiring [2–4] and transmitting [5–7] sexually transmitted infections. However, gaining a comprehensive picture of the microbiota associated with different body sites has only become possible with the development of molecular methods which are able to detect those bacteria that cannot be cultured by standard techniques and would otherwise have gone undetected [1]. Molecular methods have shown that the vaginal microbiota often contains bacteria that were missed in culture-based studies, including *Lactobacillus iners*, which is the most common vaginal *Lactobacillus* species in women of African descent [8], and bacterial vaginosis-associated bacteria including BVAB1, BVAB2 and *Mageeibacillus indolicus* (BVAB3) [9].

Currently, the most sophisticated molecular technique used to characterise the microbiota at different body sites is based on sequencing of all or part of a universally present bacterial gene, most commonly a region of the 16S rRNA gene [10]. The sequences obtained from these studies can then be used to identify the bacterial taxa present in the original sample. In order to produce a sample of bacterial DNA that can be analysed by the sequencer, the bacterial cells must first be lysed to release genomic DNA which is then purified and used to produce amplicons of the desired region of the 16S rRNA gene by PCR. The goal of this process is to produce a pool of 16S rRNA amplicons in proportions that reflect those in the original sample. However, all of the steps involved in DNA extraction and amplification may potentially bias the results of microbiota analysis [11,12].

Studies on the vaginal microbiota most commonly use a commercially available DNA extraction kit [13–17] but these methods have been poorly validated for studies on the human microbiota, and the choice of kit is often arbitrary. Commercial kits use a combination of different techniques to lyse cells, including mechanical (usually bead beating), chemical and enzymatic lysis and heating. Methods that include a bead beating step have the advantage that they concurrently homogenise the sample, but this can shear the DNA into short fragments and may increase the risk of contamination during processing [18,19]. Methods using chemical and enzymatic lysis are less likely to damage DNA, but are thought to increase the potential for extraction bias [18]. Although previous studies have compared different DNA extraction kits for microbiota analysis [18–24], the compared techniques varied considerably. It is therefore not readily evident which processes are important to ensure extracted DNA is representative of the original community. Additionally, modifications recommended by the manufacturer for pretreatment of samples containing Gram-positive bacteria are inconsistently used, making it difficult to accurately evaluate particular commercial kits for microbiota analysis. Using cultured mock communities of a mixture of eleven different human-associated bacterial species, Yuan and colleagues found that different lysis and extraction methods could alter the resulting community profile from that expected. The difference was lower for methods involving a lysis step employing either bead beating or enzymatic lysis with mutanolysin when compared to methods using neither [20].

In this study we used natural vaginal bacterial communities sampled by cervicovaginal lavage to determine whether different pretreatment lysis methods result in significant differences in DNA yield, observed taxa and community structure. We selected a variety of vaginal bacterial communities based on previous microarray profiles, in order to represent the complexity and richness of real vaginal communities. Using a commercial DNA extraction kit (Qiagen DNeasy Blood and Tissue kit) that has been used for DNA extraction from vaginal samples both in our laboratory and in previously published studies [17], we determined whether the addition of bead beating or additional lytic enzymes could alter the obtained microbiota profiles. The aim was to determine whether different lysis techniques have an impact on the results of studies on the vaginal microbiota that could alter the conclusions of individual studies or make different studies difficult to compare.

## Methods

### Sample Characteristics

The 18 cervicovaginal lavage samples used here were a subset of anonymised samples that had been collected in Rwanda as part of a study that aimed to determine whether there was an association between the type of vaginal bacterial community and prevalent infection with sexually transmitted viral diseases [25]. Ethical approval was obtained from the Rwanda National Ethics Committee and the Columbia University Medical Centre Review Board. The purpose of the current study was to evaluate lysis procedures, and samples from this study were chosen solely because the bacterial communities in these samples had previously been well-characterised by microarray analysis. We did not have access to personal identifiers and did not use any other data from the study. The 18 samples were chosen to be representative of the community clusters identified previously, including both low diversity communities dominated by either *Lactobacillus crispatus* or *Lactobacillus iners* and high diversity communities containing a mixture of strict and facultative anaerobes. Samples were stored at -80°C until analysis.

### Lysis methods

**Fig 1:**
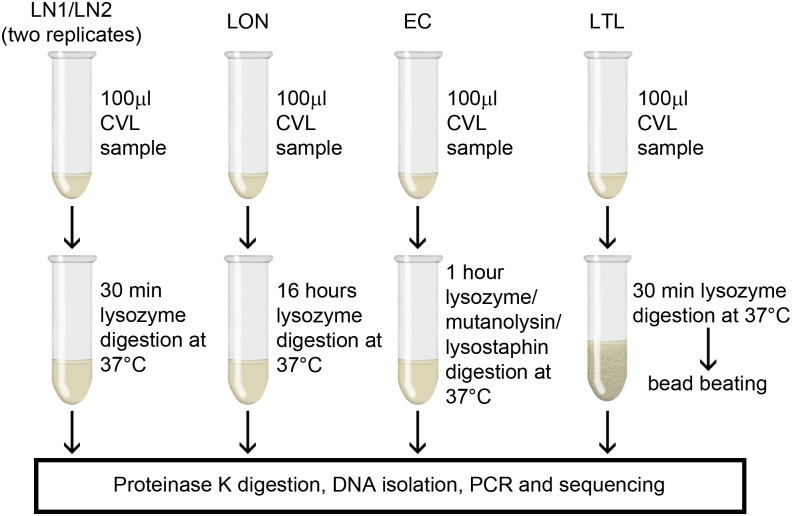
Overview of experimental design. Schematic showing how samples were processed for 16S rRNA amplicon sequencing.

To test for differences in the results of microbiota analyses resulting from different pretreatment lysis strategies, samples were thoroughly mixed by vortexing before dividing into 5 aliquots of 100 μl each and processed using one of four different lysis protocols (Fig 1). Vaginal samples may contain viscous mucoid material and if this was the case, any such material was discarded prior to vortex mixing. Two aliquots (designated "LN1" and "LN2") were subjected to 30 min of lysis at 37°C using enzymatic lysis buffer containing lysozyme from chicken egg white (20mg/ml; Sigma-Aldrich, Dorset, UK). This corresponds to the recommended pretreatment for Gram-positive bacteria as per the Qiagen DNeasy Blood and Tissue kit Handbook (Qiagen, Manchester, UK). One aliquot (designated "LON") was subjected to 16 hours of extended lysis at 37°C using enzymatic lysis buffer containing lysozyme (20 mg/ml). One aliquot (designated "EC") was subjected to 60 min of lysis at 37°C using enzymatic lysis buffer containing lysozyme (20mg/ml), mutanolysin (250U/ml; Sigma-Aldrich) and lysostaphin (22 U/ml; Sigma-Aldrich). The last aliquot (designated "LTL") was subjected to 30 min of lysis at 37°C using enzymatic lysis buffer containing lysozyme (20 mg/ml), followed by 30 s mechanical lysis at 25 Hz using 200 mg of 0.1-mm-diameter zirconia/silica beads in the Tissue Lyser II (Qiagen, Manchester, UK).

### DNA Extraction

Proteinase K and Buffer AL from the Qiagen DNeasy Blood and Tissue kit (Qiagen) were added to all aliquots before incubation at 56°C for 30 min which was followed by the remaining steps in the kit's spin column protocol, in accordance with the manufacturer's instructions and DNA was eluted in 75 μl of elution buffer. Lysis and DNA extraction was completed for all aliquots within a period of 36 hours using a previously unopened extraction kit and all work was carried out by the same person. The genomic DNA concentration of extracts was determined using the Qubit Fluorometer with the dsDNA HS Assay kit (Invitrogen Life Technologies, Paisley, UK).

### DNA Sequencing and Bioinformatics

The V3–V4 region of the 16S rRNA gene was amplified in a 25 μl reaction containing 10 ng of genomic DNA, 12.5 μl of NEBNext^®^ High-Fidelity 2x PCR Master Mix (New England Biolabs, Hitchin, UK) and 1.25 μl each of a 10 μM concentration of the conserved bacterial 16S rRNA primers 319F 5'-ACTCCTACGGGAGGCAGCAG-3' and 806R 5'-GGACTACHVGGGTWTCTAAT-3' [26] adapted with linker regions to allow barcoding of sequences using a dual-indexing approach [27]. For the first PCR (16S rRNA gene amplification) the samples were initially denatured at 98°C for 30 s, followed by 10 cycles of 98°C for 15 s, 58°C for 15 s and 72°C for 15 s, with a final extension at 72°C for 60 s. The PCR products were then purified using SeraPure magnetic beads [28], before undergoing a second PCR to attach sample specific barcodes and further amplify the region of interest. The second PCR consisted of a 25 μl reaction containing 10.5 μl of clean PCR product, 12.5 μl of 2x Ready PCR Mix and 1 μl each of a 3 μM concentration of the Illumina specific barcoding primers with the standard Illumina Nextera 8-nt index sequences. Samples were initially denatured at 95°C for 2 min, followed by 15 cycles of 98°C for 20 s, 55°C for 15 s and 72°C for 40 s, with a final extension at 72°C for 60 s. PCR products were purified, quantified using the Qubit Fluorometer with the dsDNA HS Assay kit, pooled and sequenced on the Illumina MiSeq platform (Illumina, San Diego, CA).

Sequencing reads were demultiplexed and trimmed for the presence of Illumina adapter sequences and low quality bases (quality threshold Q = 20) using Cutadapt v. 1.2.1 [29] and Sickle v. 1.200 (github.com/najoshi/sickle), respectively. The resulting reads were error corrected using SPAdes v 3.1.0 [30] and paired-end alignment was performed using PANDAseq v. 2.4 [31]. The obtained sequences were then binned into operational taxonomic units (OTUs) based on 97% sequence similarity using USEARCH v. 5.2.236 [32] through Quantitative Insights Into Microbial Ecology (QIIME v. 1.7.0)[33]. Taxonomic assignment of representative sequences (most abundant) was carried out for each OTU by comparison against the Greengenes 12_10 database in QIIME and assignments were corrected manually by NCBI BLAST search [34] for all the most abundant OTUs (≥1% in at least one extract).

### Data analysis

Calculation of alpha and beta diversity measures, hierarchical clustering and statistical analyses were performed in R version 3.2.2 [35] and using the vegan package version 2.3–1 [36]. Observed OTUs and the Simpson Index (1-D) were calculated to assess differences in alpha diversity. Hypothesis testing relating to DNA yield and alpha diversity was performed using repeated measures analysis of variance (ANOVA), correcting for differences due to the sample being extracted. Significant results were followed by pairwise comparisons using the paired *t*-test (with p-values adjusted using standard Bonferroni correction). Bray-Curtis dissimilarity and its complement, Bray-Curtis similarity, were used to report and assess differences in beta diversity. Permutational multivariate ANOVA (PERMANOVA) [37] was used to assess differences in beta diversity between different lysis methods. Hierarchical clustering was performed using the Unweighted Pair Group Method with Arithmetic Mean (UPGMA) on the Bray-Curtis dissimilarity matrix. The OTU heatmap and the principal coordinate plot were generated in R version 3.2.2 using the phyloseq package version 1.14.0 [38].

## Results

A total of 10,374,312 16S rRNA sequence reads were obtained from the 90 cervicovaginal lavage sample extracts (18 samples x 5 extractions). After quality control, paired-end alignment and assignment to OTUs, a total of 7,742,774 reads were retained, with a mean read count of 86,031 reads per sample ranging from 5,351 to 136,649 reads. The lowest read count (5,351 reads) was found to be sufficient to accurately describe sample diversity based on rarefaction curves of commonly used measures of community richness (chao1, Faith’s phylogenetic diversity index and observed OTUs), and unless otherwise stated all further analyses were carried out after samples were rarefied to 5,351 reads. A total of 549 OTUs were identified, of which 49 were present at 1% or more in at least one sample extract. Positive and negative controls were included in the sequencing run. The main contaminant present in the profiles of all the negative DNA extraction controls was a *Rhodanobacter* sp. (9.4–63.5%). This OTU was absent from the negative PCR control and has therefore most likely originated from the DNA extraction kit. Abundance of this OTU was below 0.04% in all sample extracts, indicating that contaminants originating from the extraction and amplification process were negligible in this study.

### Effect on DNA Yield

**Fig 2:**
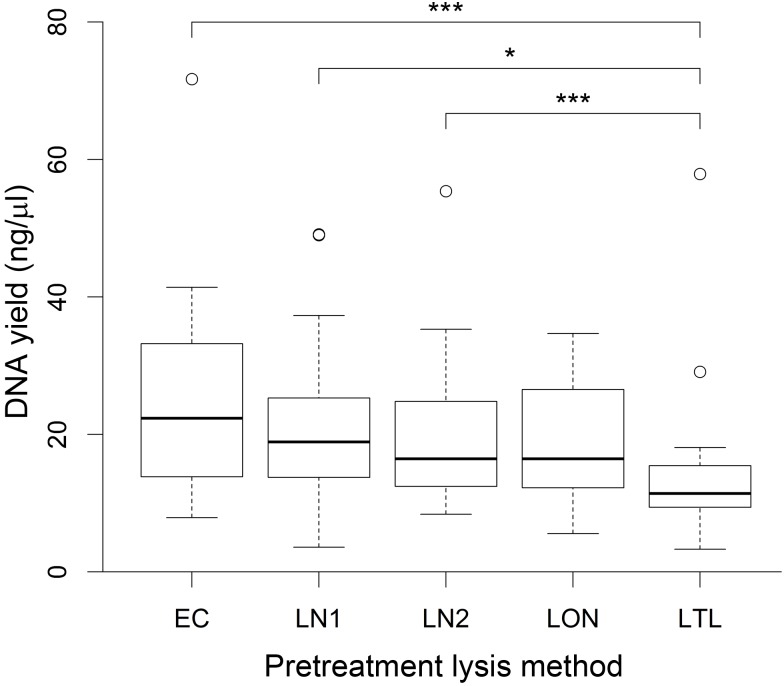
Box and whisker plot showing DNA yield obtained by each pretreatment lysis method. Boxes extend from the lower quartiles to the upper quartiles with median values indicated by the line within each box. Whiskers represent maximum and minimum values, excluding any outliers (values indicated by circles which lie outside 1.5 times the interquartile range). Significant differences between methods are starred (* P <0.05; ** P ≤0.01; *** P ≤0.001).

Four different methods for the pretreatment lysis of bacterial cells in 18 cervicovaginal lavage samples from different women were used in this study (Fig 1). Following extraction of DNA using a commercial kit, the total yield of genomic DNA was determined and compared between different lysis methods. The mean DNA yield was highest for samples extracted using the enzyme cocktail (method EC; containing lysozyme, mutanolysin and lysostaphin) and lowest for samples extracted using enzymatic lysis with lysozyme only followed by mechanical lysis (method LTL; Fig 2). Since the input volume of sample used was equal in every extraction, the measured DNA concentration can be used to compare total genomic DNA yield obtained by each method. Repeated measures ANOVA showed that there was a significant difference in the DNA concentration obtained using the four different lysis methods (P <0.0001). Pairwise comparisons showed that enzymatic lysis with lysozyme combined with bead beating (LTL) produced a significantly lower DNA yield than lysis with the enzyme cocktail (EC; P = 0.0001) or 30 min lysis with lysozyme only (P = 0.014 and P = 0.00046, for replicate runs LN1 and LN2, respectively). All other comparisons were not statistically significant at a significance level of 0.05.

### Vaginal Bacterial Community Composition

**Fig 3:**
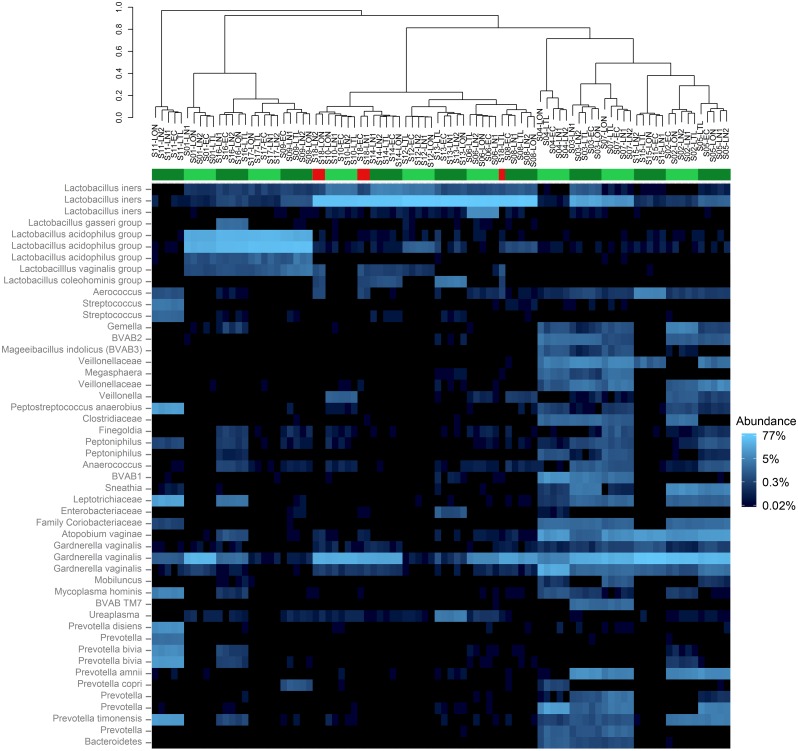
Heat map showing most abundant operational taxonomic units (OTUs) with sample extracts arranged by hierarchical clustering. All OTUs that were present at 1% or higher in at least one sample are shown. Extracts are named according to the sample of origin followed by the pretreatment lysis method used and are arranged by Unweighted Pair Group Method with Arithmetic Mean (UPGMA) clustering on the Bray-Curtis dissimilarity matrix. The coloured bar indicates which extracts have clustered most closely with all other extracts from the same sample (green) and those that have not (red). Reads have been assigned to OTUs based on 97% sequence similarity of the V3–V4 region. Note that in some cases this has resulted in multiple OTUs with the same taxonomic species identifier, which is most likely due to a high degree of intraspecies variability in this region of the gene, or incorrect base calling. *Lactobacillus* species that could not be identified to species level at the 97% cut-off have been assigned to genus subgroups: *L*. *gasseri* group (including *L*. *gasseri* and *L*. *johnsonii*), *L*. *acidophilus* group (including *L*. *acidophilus*, *L*. *helveticus*, *L*. *gallinarum*, *L*. *crispatus*, *L*. *jensenii* and *L*. *delbruekii*), *L*. *vaginalis* group (including *L*. *vaginalis* and *L*. *reuteri*) and *L*. *coleohominis* group (including *L*. *coleohominis* and *L*. *pontis*).

The samples extracted in this study had been selected to represent a variety of microbiota profiles based on previously obtained microarray data [25]. As expected, bacterial community profiles obtained for each extract in this study were either low in bacterial diversity (dominated by either *Lactobacillus crispatus* or *Lactobacillus iners)*, or high in bacterial diversity (containing a mixture of strict and facultative anaerobes including *Gardnerella vaginalis*, *Atopobium vaginae*, *Prevotella*, *Aerococcus*, *Anaerococcus*, *Streptococcus*, *Sneathia*, *Ureaplasma*, *Megasphaera*, *Mycoplasma*, *Gemella* and the bacterial vaginosis associated bacteria BVAB1, BVAB2, *Mageeibacillus indolicus* (BVAB3) and BVAB TM7) (Fig 3).

### Effect on Observed Alpha Diversity

The presence or absence of OTUs was consistent between extracts of the same sample, with any discrepancies arising due to low abundance OTUs that made up no more than 0.3% of any extract, and in 97% of those cases made up less than 0.1% of the sample. There was no statistically significant difference in the number of observed OTUs between different lysis methods (repeated measures ANOVA; P = 0.47). Calculation of the Simpson Index (1-D) confirmed a wide range of diversities (range = 0.11–0.88). Furthermore, the degree of variation between extracts from the same sample was small (maximum difference between extracts = 0.12). There was no statistically significant difference in the Simpson Index between the different methods (repeated measures ANOVA; P = 0.082).

### Effect on Observed Beta Diversity

Between extract diversity was calculated using Bray-Curtis similarity and ranged from 79.3–99.5% within samples and from 0.1–97.9% between samples. The mean difference between replicate extractions LN1 and LN2 was 4.7% (range 0.7–11.2%). Differences between extracts from the same sample were due to differences in proportions of observed OTUs, rather than differences in the presence/absence of OTUs. There was a negative correlation between the minimum within-sample Bray-Curtis similarity and the mean number of observed OTUs for that sample (Spearman’s rank correlation: r = -0.62; P = 0.007). In other words, samples with higher OTU richness tended to have increased dissimilarity between extracts.

**Fig 4:**
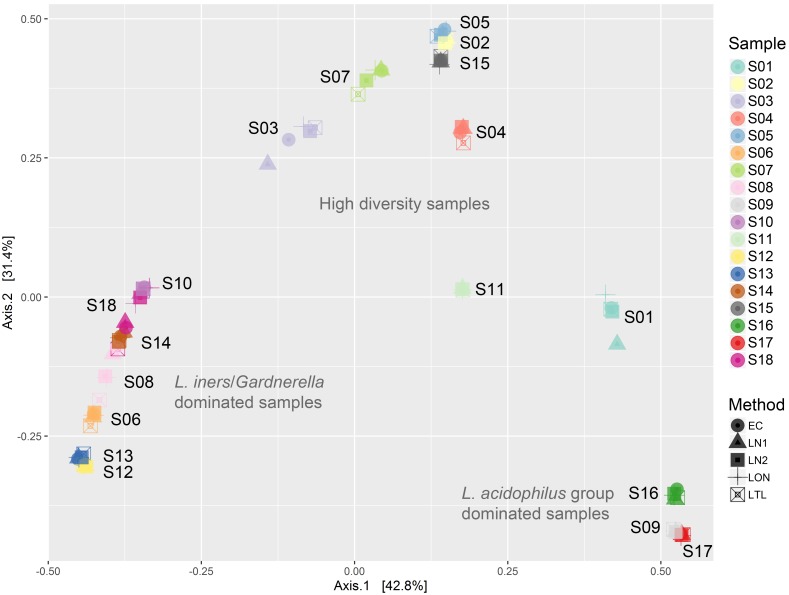
Principal coordinate analysis ordination of a Bray-Curtis dissimilarity matrix. Extracts are coloured by sample of origin. Extracts cluster closely with other extracts originating from the same sample and there is no observable effect of pretreatment lysis method. Extracts from samples that are dominated by *Lactobacillus iners* with variable proportions of *Gardnerella* have clustered on the left, extracts from samples that are dominated by *L*. *acidophilus* group have clustered on the bottom right and extracts from high diversity samples that contained a mixture of strict and facultative anaerobes cluster towards the top.

PERMANOVA analysis of Bray-Curtis dissimilarity showed that the differences between extracts originating from different samples (R^2^ = 0.99, P = 0.001) were far greater than differences between different lysis methods (R^2^ = 0.00086, P = 0.029). Although the effect of lysis method was significant in this analysis, the magnitude of this effect was negligible when compared to the differences due to the sample of origin. This is reflected in the hierarchical clustering of the extracts based on Bray-Curtis dissimilarity scores (Fig 3) and the clustering of extracts by principal coordinate analysis ordination of the Bray-Curtis dissimilarity matrix (Fig 4), which resulted in clustering of the extracts by sample rather than lysis method. Pairwise comparisons revealed that the greatest differences were between methods LON and LTL (PERMANOVA; R^2^ = 0.0016, unadjusted P value = 0.044) and LON and LN1 (PERMANOVA; R^2^ = 0.00091, unadjusted P value = 0.046), but these differences were not statistically significant after adjustment for multiple testing (standard Bonferroni correction).

The largest cluster of extracts was composed of samples that were dominated by *L*. *iners* (66–97%) with a variable proportion of *G*. *vaginalis* (0–31%). In this group, one set of extracts (from sample S18) clustered more closely with extracts from other samples than with each other, as a result of higher Bray-Curtis similarity with extracts of other samples. This is due to small differences in observed proportions of OTUs and has occurred because of the high degree of similarity between the seven samples in this cluster. The Bray-Curtis similarity score ranged from 65.3–97.9% between extracts from different samples in this group. Since the composition of these samples was similar, we repeated the PERMANOVA analysis on this subset alone to minimise any effect of differences in alpha diversity on the magnitude of beta diversity scores. In this analysis, the differences due to sample remained highly statistically significant (R^2^ = 0.96, P = 0.001), but differences between different lysis methods did not (R^2^ = 0.0059, P = 0.38).

### Effect on Individual OTUs

Certain bacterial species have previously been reported to be resistant to lysozyme, including *Neisseria gonorrhoeae* [39] and staphylococci [40]. Furthermore, the results of a recent study indicate that streptococci may be underestimated in microbiota analyses [11]. In order to investigate whether different lysis methods influenced the proportions of these bacteria, OTUs assigned to these taxa were identified and compared between different methods. Since these taxa were present at very low levels, calculations were performed on proportions calculated from raw read counts (i.e. prior to rarefaction). One OTU identified in this study was assigned to the genus *Neisseria*. This could not be identified to species level due to 100% sequence similarity of related species in this region of the 16S rRNA gene, but is most likely to represent *N*. *gonorrhoeae* in this niche. This OTU was present at a low level (≤0.18% and no more than 185 reads per extract) in extracts from 5 different samples. All of the extracts from these samples contained reads mapping to this OTU, with the exception of the sample with the lowest abundance of this OTU (≤0.003%), where there were no reads in the extract that had been lysed with lysozyme overnight. There was no statistically significant difference between the percentage of this OTU between different lysis methods (repeated measures ANOVA; P = 0.54). A further OTU identified as a *Staphylococcus* sp. was present at a very low level (≤0.06% and no more than 55 reads per extract), and was consistently present in extracts from the 4 samples for which the proportion was >0.01%. 11 OTUs mapped to *Streptococcus* spp, of which only 2 totalled >1000 reads. These were consistently present in one sample (ranging from 1.4–2.1% and 2.3–3.7% with a maximum of 2424 and 4063 reads in a single extract), with all remaining extracts containing less than <0.05% of either OTU. There was no statistically significant difference between the percentage of this OTU between different lysis methods for either genus (repeated measures ANOVA; P ≥ 0.3).

## Discussion

Previous studies have used mock communities to be able to assess different lysis methods [19,20]. Mock community studies have the advantage that the community profile of the samples is known, allowing assessment of the accuracy of the results [20]. In this study, we chose to use naturally occurring bacterial communities for which there is no gold standard measure of community composition [41]. The true composition of the samples in this study is therefore not known and as a result the accuracy of each lysis method cannot be determined. However, using biological samples that cover a range of different community types has the advantage of allowing comparison of lysis methods on a wider range of bacteria commonly encountered in the vaginal niche, including those that have not or have rarely been cultured. This includes the bacterial vaginosis-associated bacteria, which can make up a substantial proportion of the bacterial population in some individuals [42]. Additionally, using vaginal samples allowed us to compare the magnitude of the effect of method with that resulting from biological differences between samples from different individuals. It should also be noted that vaginal samples can vary in consistency [43] and may contain viscous mucoid material that is difficult to homogenise. In this study, we have chosen to remove any such material where present prior to processing to minimise any potential variation between extracts resulting from inadequate homogenisation. It is possible that the composition of the microbiota associated with the removed material differed from the remaining material and could therefore have changed the overall profile of the samples.

The results of this study clearly show that sample has a far greater effect on the microbiota profile than the pretreatment lysis method. This is consistent with the results of studies that have compared different extraction kits or protocols for faecal samples [18,21,23] and saliva [22]. Additionally, epidemiological studies investigating the effect of vaginal bacterial communities on health commonly group samples by clustering based on overall community structure, assigning each sample to a “community type” [25,44–47] and accurate clustering of extracts was not affected by pretreatment lysis method in this study. However, biological differences resulting from subtle variation in proportions of taxa may be difficult to separate from experimental variation as evidenced by up to 11.2% dissimilarity between replicate extracts LN1 and LN2, and should therefore be interpreted with caution. A larger sample size and greater number of experimental replicates would be required to investigate this variation further, particularly to determine whether the other lysis methods used in this study would produce a similar degree of dissimilarity.

In this study, we used the recommended protocol for the pretreatment of Gram-positive bacteria with lysozyme as standard since it is thought to improve species representation [19]. As a result, we cannot make any conclusions about the necessity of this enzyme for bacterial lysis from the data in this study. Lysozyme breaks down the bacterial cell wall by cleaving peptidoglycan and may be particularly important for the breakdown of the thick peptidoglycan layer of the Gram-positive cell wall [48]. However, modifications to peptidoglycan structure can render bacteria resistant to lysozyme digestion. This has been reported in *N*. *gonorrhoeae* [39] and staphylococci [40], both of which could be present in vaginal samples [49,50]. The use of the enzymes mutanolysin and lysostaphin in addition to lysozyme has been recommended for the lysis of vaginal samples in order to lyse bacterial species that are resistant to lysozyme digestion [20]. Lysostaphin specifically lyses some *Staphylococcus* spp. [40] and mutanolysin is active against the cell wall of some streptococci [51]. In this study, we failed to identify any differences between lysis methods for the aforementioned bacterial taxa. However, the number of 16S rRNA reads mapping to these genera was small, resulting in low statistical power to detect relatively small differences. Additionally, the bacterial species/strains sequenced in this study may not have been resistant to lysozyme lysis. For example, differences in susceptibility to lysozyme digestion between different strains of *N*. *gonorrhoeae* have been reported [39]. It is possible that differences in lysis efficiency may have been evident if different species or strains had been present in the samples used. However, the addition of mutanolysin and/or lysostaphin to samples in this study, which contained the majority of major vaginal bacterial taxa, was not found to significantly alter the presence/absence of OTUs or their relative abundance. It is therefore unlikely that the addition of these enzymes would alter the conclusions of studies designed to investigate the impact of vaginal community type on human health.

A further additional treatment that can be used to improve lysis of cells is mechanical disruption, usually by bead-beating. Bead beating has been reported to increase the observed richness in previous microbiota studies [18,52]. This was not the case in this study in which we found no significant difference in alpha diversity. It should be noted that bead-beating may have a greater influence on fresh samples compared with those that have been stored in the freezer, possibly due to disruption of the Gram-positive cell wall by freeze-thawing [23]. The samples used in this study were stored at -80°C, as is common for vaginal microbiota studies [13,44,47,53–61] and it is possible that an effect of bead beating would have been evident if fresh samples had been used, by resulting in reduced richness in those extracts that were not subjected to bead beating.

In contrast to the effect on diversity, we found that the addition of a bead-beating step significantly reduced the DNA concentration of the extract, which is consistent with previous results using mock bacterial communities [19,20], and is most likely due to some material being lost with the beads when they are removed from the sample. DNA yield is commonly used to assess the efficiency of different lysis and extraction protocols. Other studies have reported that the inclusion of a bead-beating step led to an increase in DNA yield from activated sludge [52] and faecal samples [24]. However, these samples may be more heterogeneous and particulate in nature, which could explain this difference and emphasises the importance of validating methods for microbiota analysis on the sample type of interest. It should be noted that DNA extraction in this study was carried out with the same commercial kit (Qiagen DNeasy Blood and Tissue) for all samples. Proprietary extraction kits employ a variety of different techniques to lyse cells and purify DNA. Hence the importance of pre-treatment with additional lysis methods may vary between kits.

In the current study, we aimed to assess the impact of lysis method on the observed microbiota profile, since this is the main outcome measure in many recent studies of the human microbiota, including the vagina [25,44–47]. It is therefore interesting to note that although we observed a significantly lower DNA yield when mechanical lysis was added to the protocol, this could not be associated with any differences in the observed richness or evenness of OTUs. However, recent work has highlighted that, in studies using next-generation sequencing technology to characterise the bacterial microbiota, low amounts of template DNA are associated with a proportional increase in contaminant taxa originating from laboratory reagents [62–64]. These contaminants can lead to erroneous conclusions [62]. In this study, 10 ng of template DNA was used in each 25 μl PCR reaction. This amount of DNA has been found to result in significantly lower variability in microbiota community structure in studies profiling the faecal microbiota [63]. Furthermore, in our laboratory, this concentration of DNA in cervicovaginal samples resulted in negligible levels of reagent contamination (unpublished data), which is supported by the low levels of the contaminant *Rhodanobacter* OTU in this study. *Rhodanobacter* spp. have been isolated from environmental soil and water samples [65,66]. Interestingly, this genus has also been reported as a member of the human microbiota [67], highlighting the need to adequately control for contamination occurring during laboratory processing of samples for 16S rRNA microbiota profiling [62]. Since all of the methods used in this study produced sufficient DNA to avoid significant contamination arising during processing, and higher DNA yield has not been shown to be associated with improved accuracy of microbiota profiles in mock community studies [19,20], the reduced DNA yield with method LTL is not of particular concern. However, increased contamination caused by inclusion of a bead-beating step has been reported [19] and may be best avoided in the absence of a clear advantage.

## Conclusions

It is widely acknowledged that bias exists in 16S rRNA studies describing microbiota profiles and that no currently available method is able to perfectly describe the community being analysed [10]. However, an understanding of how the choice of laboratory methods affects the results of such studies is important in order to accurately interpret the results and make valid comparisons between different studies. Although we were able to identify significant differences in DNA yield and diversity between the different methods used in this study, the effects of this were much smaller than those due to the sample and did not alter the grouping of extracts by hierarchical clustering and principal coordinate analysis. However, since there was an observable effect of lysis method on microbiota composition, we recommend that the same method is used within a study to reduce the risk of introducing a differential bias. Furthermore, comparisons between studies using different lysis methods should be made with care, but will likely be of much smaller magnitude than differences caused by the choice of extraction kit and 16S rRNA primers [12]. Additionally, studies with a focus on the abundance of a particular bacterial species should include additional techniques such as qPCR to confidently identify any differences between groups.
